# An Interesting Case of Balloon-Assisted Deployment of a Dislodged Coronary Stent

**DOI:** 10.7759/cureus.55257

**Published:** 2024-02-29

**Authors:** Dibyasundar Mahanta, Anoop Kumar Singh, Suresh Kumar Behera, Debasish Das

**Affiliations:** 1 Cardiology, SUM Hospital, Bhubaneswar, IND; 2 Cardiology, Institute of Medical Sciences & SUM Hospital, SOA University, Bhubaneswar, IND; 3 Cardiology, All India Institute of Medical Sciences, Bhubaneswar, IND

**Keywords:** stent, coronary, dislodged, assisted, balloon

## Abstract

We report a first and interesting case of balloon-assisted deployment of a dislodged coronary stent. While performing a calcified left circumflex coronary artery (LCX) intervention, the drug-eluting coronary stent was dislodged in the osteoproximal segment of the calcified and tortuous LCX. The dislodged stent was rewired, progressively dilated with multiple balloons and, finally, a larger, balloon-mounted stent was pushed forward, positioned across the coronary lesion and deployed, resulting in distal thrombolysis in myocardial infarction (TIMI) III flow with good angiographic results. Rewiring the dislodged stent with subsequent balloon-assisted deployment in the lesion can be a solution for a dislodged coronary stent in the proximal vessel.

## Introduction

Stent dislodgement inside the coronary artery may serve as a catastrophe as it may lead to abrupt vessel closure, ventricular arrhythmia, and sudden cardiac death [1]. The conventional solution to stent dislodgement includes retrieval of the dislodged coronary stent and crushing the stent across the segment to prevent distal embolization. [1] We here describe a novel technique of management of a dislodged coronary stent in which the dislodged stent was rewired, a balloon was inflated inside the stent at high atmospheric pressure, and the balloon-stent assembly was pushed forward so that it could be deployed across the desired lesion segment, which can be called as '' balloon-assisted deployment of the dislodged coronary stent". The present novel technique could avoid the disadvantage of putting an additional layer of metals across the osteoproximal segment of a coronary artery by conventional crushing of the coronary stent. Lesser the metal, better the outcome of coronary intervention as it is associated with a lesser incidence of stent thrombosis and in-stent restenosis. The balloon-assisted technique converts a dislodged coronary stent into a conventional stent with a balloon inside but it requires quiet patience in achieving progressive balloon dilatation to create a better lumen inside the coronary stent with subsequent deployment. Tortuosity and dense calcium are the two known enemies of successful stent deployment. Stent dislodgement can be successfully managed with this novel balloon-assisted technique. Stent dislodgement with subsequent embolization to the cerebral circulation results in ischemic stroke, but stent embolization into the peripheral circulation may behave innocent. Paradoxically, stent dislodgement inside the coronary artery needs an urgent catheter solution, either in the form of balloon-assisted retrieval, crushing of the stent, or novel balloon-assisted deployment as described in the present case. 

## Case presentation

**Figure 1:**
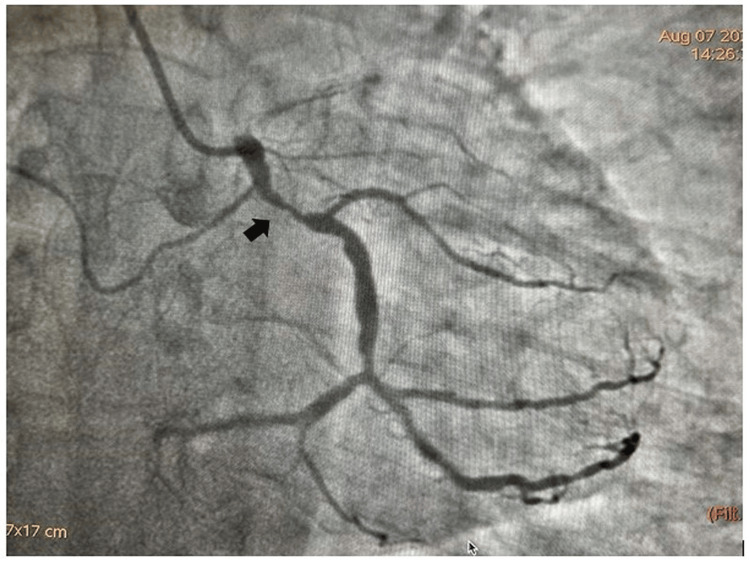
Tortuous and densely calcified lesion in proximal left circumflex coronary artery (LCX)

An 80-year-old male presented to the cardiology outpatient department with effort angina New York Heart Association (NYHA) Class II for the last six months. He had profuse diaphoresis and shortness of breath for the last week. He had no history of palpitation, presyncope or syncope in the past. During the examination, he had a blood pressure of 150/90 mmHg in the right arm supine position with a heart rate of 76 beats per minute. His cardiovascular system examination revealed the presence of left ventricular fourth heart sound (LVS4) due to a stiffer ventricle in the elderly age with the presence of associated hypertension. His serum Troponin T level was elevated (38 ng/l) and N-terminal brain Natriuretic Peptide (NT pro-BNP) was modestly elevated (650 pg/ml). His echocardiography revealed the presence of degenerative aortic sclerosis with normal left ventricular systolic function (ejection fraction=55%). He had no regional wall motion abnormality in echocardiography. He was subjected to an invasive right transradial coronary angiogram which revealed a calcific subtotal occlusion (90-95%) of mid left circumflex coronary artery (LCX) (Figure 1).

**Figure 2:**
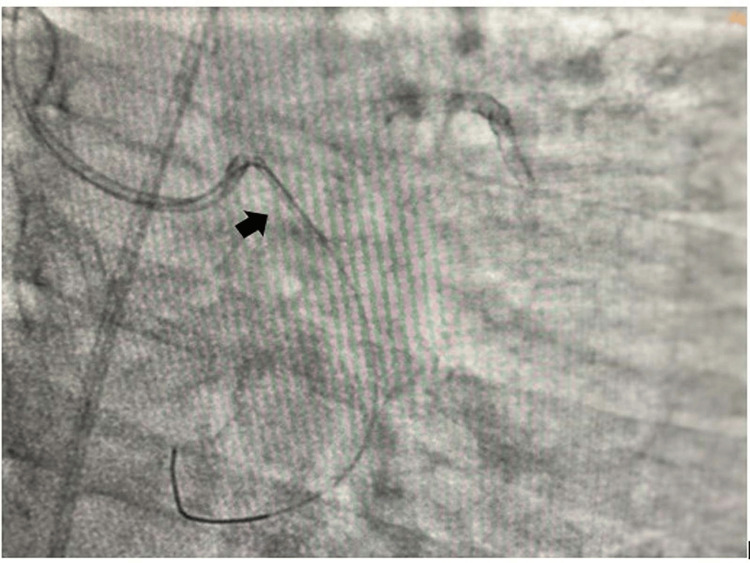
Wiring through the dislodged coronary stent in osteoproximal left circumflex coronary artery (LCX)

**Figure 3:**
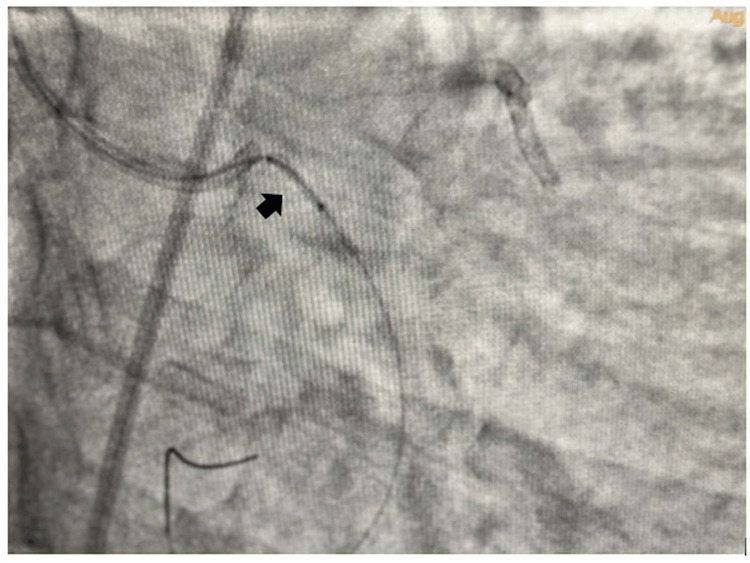
Inflation of a 1.25x8 mm semicompliant balloon across the proximal half of the dislodged coronary stent

**Figure 4:**
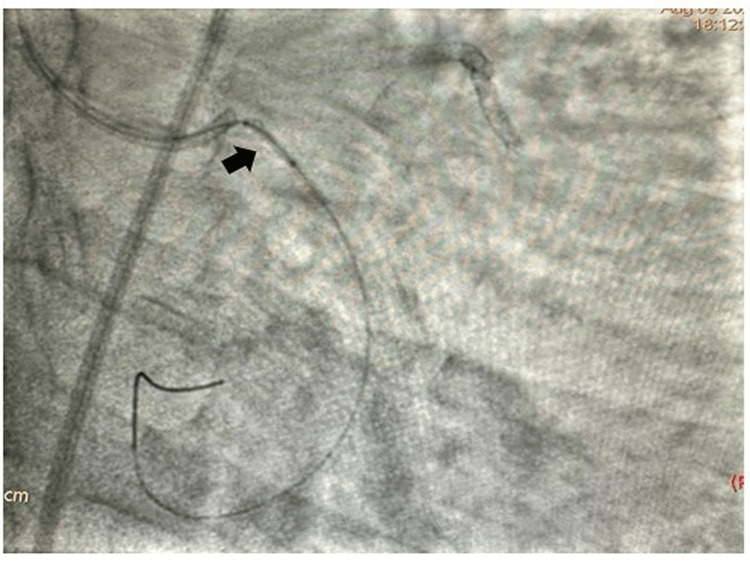
Inflation of a 1.5x6 mm semicompliant balloon inside the dislodged coronary stent

**Figure 5:**
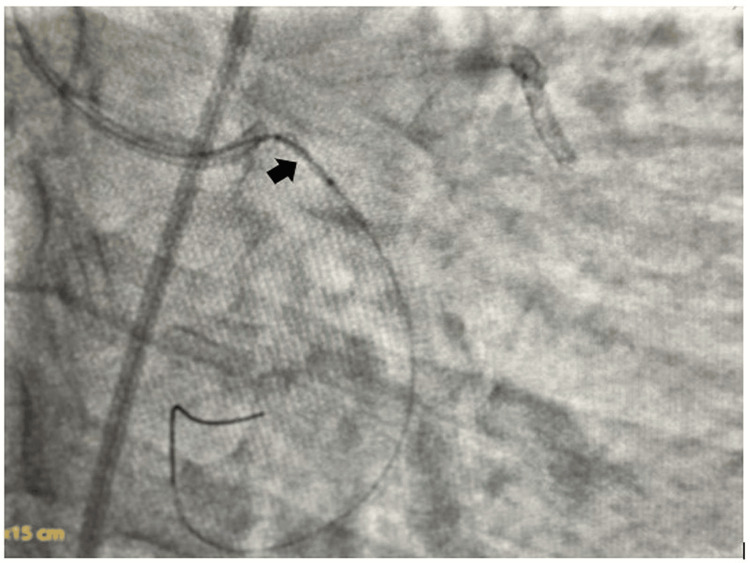
Inflation of a 2x10 mm semicompliant balloon inside the dislodged coronary stent

**Figure 6:**
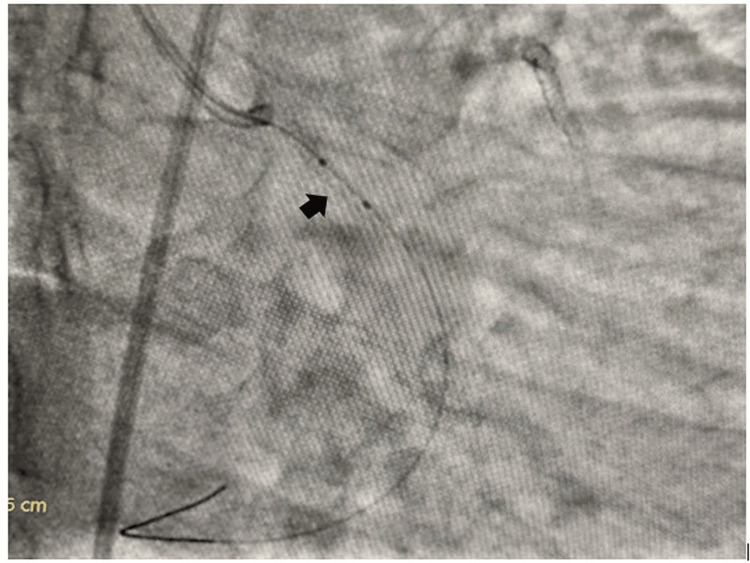
A 2x10 mm balloon mounted stent is pushed forward to deploy across the lesion (balloon-assisted deployment of dislodged coronary stent)

**Figure 7:**
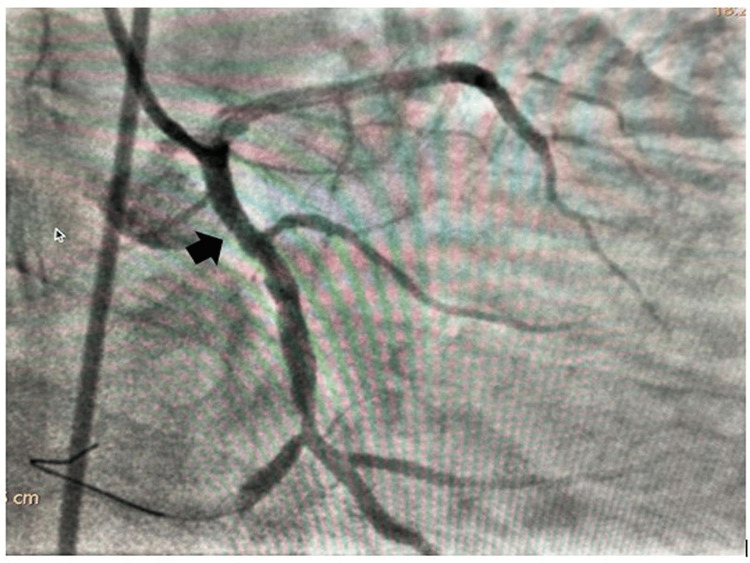
Good angiographic result post final deployment of dislodged coronary stent

The left main coronary artery was engaged with Extraback Up (EBU) 6F 3.5 guide catheter and the lesion in LCX was crossed with 0.014'' Run-through guide wire. The lesion was predilated with 2x10 mm and 2.5x10 mm semi-compliant balloons and a 3.5 x20 mm drug-eluting stent (DES) was tried to cross the lesion. The DES did not cross the lesion, for which further dilatation of the lesion with 2.75x10 mm and 3x10 mm balloons was performed. Then we tried to negotiate the DES across the lesion. While taking out the stent, the osteoproximal calcium spur caught the stent, which was dislodged at the ostium of the LCX. A 0.014 Fielder FC wire with a small tip (1 mm ) was wired across the dislodged stent (Figure 2). A small balloon 1.25x8 mm in size was inflated across the proximal half of the dislodged coronary stent as it could not cross the full length of the coronary stent (Figure 3). Then a 1.5x6 mm balloon was inflated inside the dislodged stent in order to create a larger lumen (Figure 4). Then a 2x10 mm balloon was inflated inside the stent (Figure 5) and the stent-balloon assembly was pushed forward to deploy the stent across the lesion (balloon-assisted deployment) (Figure 6). As the vessel was 3.5 mm in diameter, 2.5x10 mm and 3.5x10 mm balloons were inflated across the dislodged stent successively to deploy it across the coronary lesion. Post percutaneous transluminal coronary angioplasty (PTCA), there was good angiographic result with thrombolysis in myocardial infarction (TIMI) III flow (Figure 7). The patient was put on dual antiplatelets including ticagrelor 60 mg twice daily with beta-blocker, statin and low-molecular-weight heparin 0.4 ml subcutaneous twice daily for three days. The patient symptomatically improved and had no shortness of breath or angina. On follow-up, he was doing well after one month without any novel ischemic ST-T changes. Our case is the first demonstration of a unique technique of balloon-assisted deployment of a dislodged coronary stent which saves the patient from conventional crushing of dislodged coronary stents inside the lumen of the coronary artery. We did not crush the stent at the ostium of the LCX in order to avoid four layers of metals across the ostium of the LCX, which may invite thrombus and may make it prone to restenosis in the future.

## Discussion

The incidence of stent dislodgement is approximately 0.3% during routine coronary intervention [2]. Stent dislodgement results in complication in almost 19% of cases [2]. It may result in catastrophic complications in the form of acute stent thrombosis, cardiac arrhythmia and sudden cardiac death. When stent dislodgement into the bloodstream is predicted to be unharmful, it is left in situ in almost 3% of cases [2]. Most often, the dislodged stent in the coronary artery is crushed with a balloon to prevent distal embolization. Sometimes, the dislodged coronary stent is retrieved with a small balloon catheter, which is passed across the stent. The balloon is inflated distal to the dislodged stent, and another wire is passed beside the dislodged coronary stent. Both wires are twirled together at the distal end, and the dislodged coronary stent is retrieved. Crushing of the coronary stent produces an additional layer of metal in the coronary artery, which serves as a risk factor for acute stent thrombosis and subsequent instent restenosis. In our case, the proximal presence of tortuosity and dense calcium were the risk factors behind the stent dislodgement. Osteoproximal LCX is known as the "no touch zone" in coronary intervention. After the stent dislodgement in the osteoproximal coronary artery, we did not opt for crushing the stent in the osteoproximal segment as deploying two additional layers of metals across the osteoproximal LCX would serve as a nidus for acute stent thrombosis and in-stent restenosis in future.

We negotiated a 0.014'' guide wire across the dislodged coronary stent and then a 1.25x8 mm balloon was tried to negotiate across the stent which only traversed half a length of the dislodged stent and was inflated at 12 atm pressure there. Post balloon inflation with a 1.25x8 mm balloon, we were able to negotiate a 1.5x6 mm balloon inside the dislodged stent, which was again inflated at 12-14 atm pressure, after which we were able to negotiate a 2x10 mm semi-compliant balloon. The 2x10 mm semi-compliant balloon was dilated at 16 atm pressure and the balloon-stent assembly was pushed together forward to deploy across the lesion (balloon-assisted deployment of dislodged coronary stent). Then a 3.5x10 mm semi-compliant balloon was inflated inside the positioned stent at 16-18 atm pressure in order to achieve final deployment. Post stent deployment, there was good angiographic result with distal TIMI III flow. Small vessels, tortuous vessels, extreme angulation, dense calcification, presence of a previous stent and poor guide catheter support like Judkins Right catheter (JR) are the predictors of stent dislodgement inside the coronary circulation [3-5]. Although crushing of the coronary stents is always the last resort in dislodged coronary stents, we did not opt for the same as we thought it would be unwise to burden the osteoproximal LCX with two additional layers of metals. In the event of osteoproximal dislodgement of a coronary stent, balloon-assisted deployment of a coronary stent across the lesion serves as a novel option in preventing the untoward effects of additional layers of metal created by conventional stent crushing. 

## Conclusions

We report the first and most interesting case of balloon-assisted deployment of a dislodged coronary stent, which resulted in good angiographic results with distal TIMI III flow. Balloon-assisted deployment of a dislodged coronary stent prevents additional layers of metals in the coronary artery secondary to conventional crushing of dislodged coronary stents. Crushing of dislodged coronary stents results in an additional layer of metals which itself serves as a risk factor for stent thrombosis and instent restenosis. Balloon-assisted deployment of coronary stent serves as a novel option for deployment of dislodged coronary stent across the coronary lesion. 
